# Beyond Corticosteroids: Ultrasound-Guided Regenerative and Hydrodissection Therapies for Adhesive Capsulitis

**DOI:** 10.3390/life16081270

**Published:** 2026-07-31

**Authors:** Chih-Ya Chang, Yen-Sheng Lin, Po-Yin Chen, Li-Wei Chou

**Affiliations:** 1 Department of Physical Medicine and Rehabilitation, Tri-Service General Hospital, National Defense Medical University, Taipei 11490, Taiwan; 2 Department of Physical Medicine and Rehabilitation, School of Medicine, College of Medicine, National Defense Medical University, Taipei 11490, Taiwan; 3 Department of Physical Therapy and Assistive Technology, National Yang Ming Chiao Tung University, Taipei 112, Taiwan; poyin1128@nycu.edu.tw (P.-Y.C.); lwchou@nycu.edu.tw (L.-W.C.); 4 Graduate Institute of Medical Science, National Defense Medical University, Taipei 11490, Taiwan; johnnylin99@gmail.com

**Keywords:** adhesive capsulitis, frozen shoulder, ultrasound-guided injection, hyaluronic acid, prolotherapy, platelet-rich plasma, hydrodissection, suprascapular nerve, regenerative medicine, rehabilitation

## Abstract

Adhesive capsulitis (AC) is a common fibroinflammatory shoulder disorder, affecting 2–5% of the general population and up to 20% of people with diabetes mellitus. Although historically considered self-limiting, the course frequently exceeds two years and imposes a substantial functional burden. Intra-articular corticosteroids remain the predominant first-line option, but their benefit is often short-lived and is offset by transient hyperglycemia in diabetic patients, potential tissue toxicity with repeated use, and limited durability. These limitations have prompted interest in non-steroidal, ultrasound-guided alternatives. This narrative review appraises two complementary strategies: ultrasound-guided regenerative injectates (hyaluronic acid, hypertonic dextrose prolotherapy, and platelet-rich plasma) and ultrasound-guided perineural hydrodissection of the suprascapular, axillary, and subscapular nerves. We summarize comparative efficacy with corticosteroids, durability, injection anatomy, and the subgroups most likely to benefit. We then propose—explicitly as a clinically defensible framework rather than a validated guideline—a phase-specific paradigm in which a timely non-steroidal injection provides early pain control while stage-appropriate rehabilitation drives functional recovery. The hypothesis that this sequence may compress the symptomatic course to approximately three to six months is supported by preliminary evidence and requires prospective validation. Because no head-to-head trial has compared the proposed paradigm with the corticosteroid-first standard, these recommendations warrant corresponding caution.

## 1. Introduction

Adhesive capsulitis (AC), or frozen shoulder, is a chronic fibroinflammatory disorder of the glenohumeral joint, characterized by progressive pain and a global, capsular-pattern loss of both active and passive range of motion. It affects 2–5% of the general population and 10–20% of patients with diabetes mellitus, with even higher rates in those with poor glycemic control or insulin-dependent disease [1,2]. AC most commonly presents in adults aged 40–60 years, with a female predominance, and population-based data identify prior shoulder injury, osteoarthritis, thyroid disease, prolonged immobilization, and prior shoulder surgery as recognized associations [3]. The relationship with dyslipidemia is frequently cited but may be partly confounded by coexisting diabetes and thyroid dysfunction, and its independent causal role remains uncertain [4]. The functional burden is disproportionate to the condition’s prevalence: patients are frequently unable to dress, drive, sleep on the affected side, or manage overhead work, and upper-extremity impairment of this kind is associated with measurable work absence and reduced everyday function, particularly in people with diabetes [5].

AC has long been described as self-limiting, but this characterization is increasingly questioned by longitudinal data. Long-term cohorts indicate that a substantial proportion of patients—by some estimates 30–50%—retain measurable pain or stiffness beyond two years, and a subgroup does not recover fully without intervention [6,7]. Patients with diabetes tend to fare worse, with denser capsular fibrosis, slower resolution, and higher recurrence [1,2]. These observations provide a rationale for considering earlier intervention—particularly while the capsule remains in a predominantly inflammatory phase rather than a structurally adhesive one—rather than deferring treatment on the assumption of spontaneous resolution.

The prevailing view in the most authoritative recent syntheses remains conservative. Millar and colleagues, in *Nature Reviews Disease Primers*, place corticosteroid injection and physiotherapy at the center of management and treat regenerative options as adjuncts [8]. The *JAMA Network Open* meta-analysis by Challoumas et al. concludes that early intra-articular corticosteroids combined with home exercise gives the best short-term recovery [9], and the most recent narrative review in the *American Journal of Medicine* retains the same anchor, reserving regenerative and neuromodulation strategies for refractory cases [10]. Our reading of the emerging evidence suggests that a more active, non-steroidal-first stance may be defensible in selected patients, for three reasons. First, several head-to-head randomized trials at 12–24 weeks favor platelet-rich plasma (PRP) and hyaluronic acid (HA) over corticosteroids in pain, disability, and range of motion (ROM), although these trials are mostly single-center and of modest size [11,12,13]. Second, perineural hydrodissection adds a distinct neuromodulation axis that intra-articular pharmacotherapy cannot replicate, and it can produce analgesia rapid enough to broaden tolerance to rehabilitation [14,15]. Third, wearable and elastography technologies increasingly allow objective tracking of recovery, so individualized titration need not rest on clinical impression alone [16,17]. The intent of this more active stance is to shorten the painful trajectory and preserve autonomy in daily life, not to displace rehabilitation, which remains the principal driver of functional recovery.

Two complementary strategies define the contemporary toolbox: (i) ultrasound-guided regenerative injectates that modulate the capsular fibroinflammatory environment—HA, hypertonic dextrose prolotherapy, and PRP; and (ii) ultrasound-guided perineural hydrodissection that modulates the sensory pathways serving the glenohumeral joint—the suprascapular, axillary, and (selectively) subscapular nerves. We argue that, in appropriately selected patients, clinicians may consider a more proactive paradigm than first-line corticosteroids: an ultrasound-guided regenerative injection (HA, hypertonic dextrose, or PRP), combined where appropriate with perineural hydrodissection of the suprascapular and axillary nerves, and integrated with stage-appropriate rehabilitation. In our clinical experience this combination targets three objectives that corticosteroid monotherapy addresses less completely: faster control of disproportionate pain, prevention of the slow maturation of capsular adhesions, and earlier restoration of complex multi-planar daily life function. We emphasize at the outset that this proactive paradigm has not been tested head-to-head against the prevailing corticosteroid-first standard, and we present it as a clinically defensible framework rather than an established guideline (Table 1). The aims of this review are to summarize the limitations of corticosteroid monotherapy and the rationale for non-steroidal alternatives; to appraise the comparative efficacy and durability of HA, prolotherapy, PRP, and perineural hydrodissection; to describe the injection approaches and anatomical targets relevant to everyday practice; to identify the subgroups in whom each strategy is most defensible; to propose a phase-specific framework in which timely ultrasound-guided injection plus stage-appropriate rehabilitation may shorten recovery; and to outline the objective monitoring tools that are beginning to make recovery measurement practical in clinic.

## 2. Methods of Literature Synthesis

**Figure 1:**
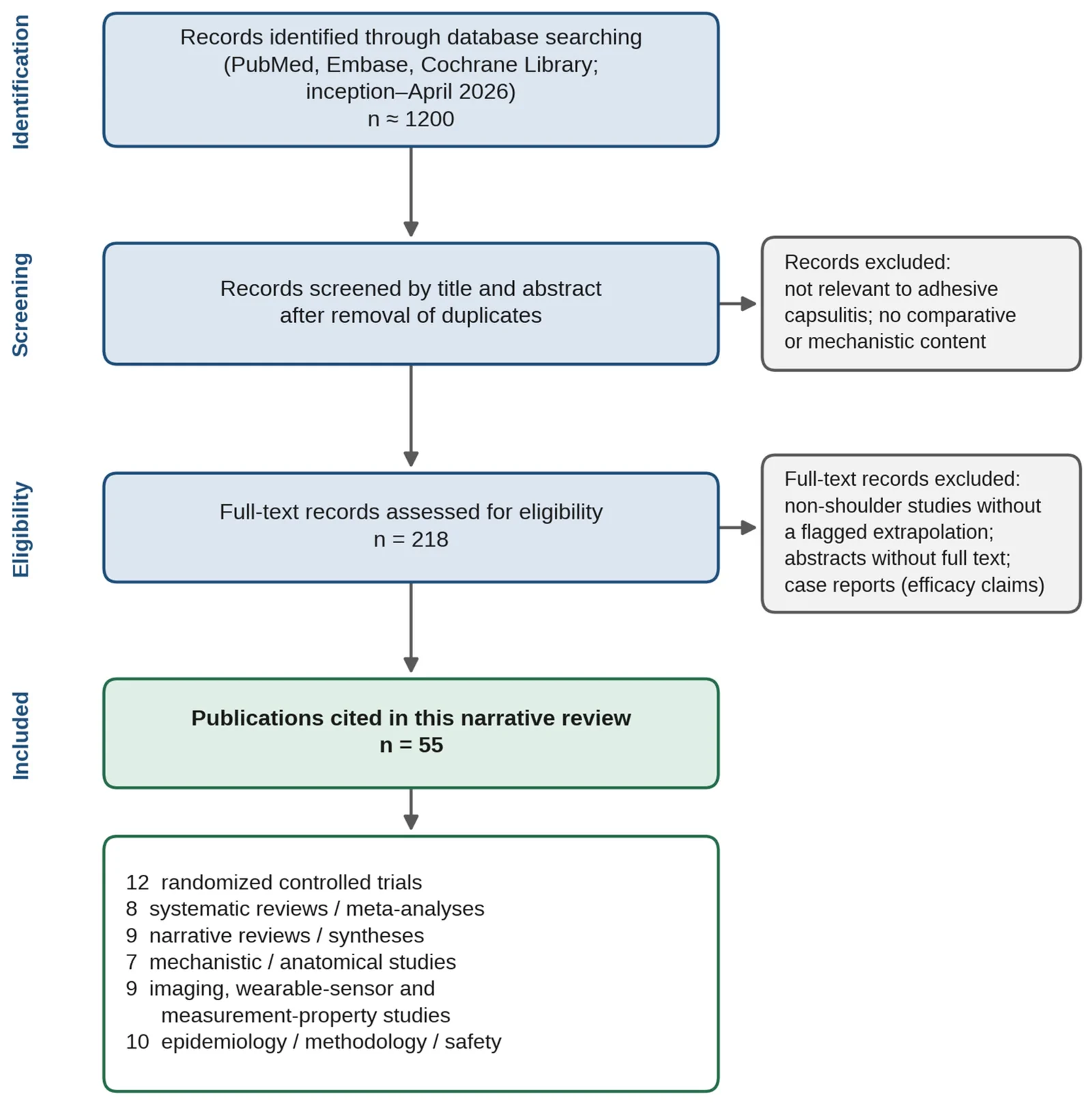
PRISMA-adapted flow diagram of the literature identification, screening, and selection process. Approximately 1200 records were identified across PubMed, Embase, and the Cochrane Library (inception–April 2026); after duplicate removal and title/abstract screening, 218 records were assessed in full text, and 55 publications were cited in this narrative review. The PRISMA framework [18] is applied here to improve the transparency of the selection and is not intended to imply the completeness of a systematic review.

This is a narrative review; it does not claim the methodological completeness of a systematic review, and its conclusions should be interpreted accordingly. To improve transparency, we report the search and selection process in a structured form and provide a flow diagram (Figure 1) adapted from the PRISMA 2020 framework [18], recognizing that PRISMA is designed for systematic reviews and is used here only to make our selection process auditable.

**Search strategy.** We searched PubMed, Embase, and the Cochrane Library from database inception through April 2026, using combinations of the terms *adhesive capsulitis*, *frozen shoulder*, *hyaluronic acid*, *prolotherapy*, *dextrose*, *platelet-rich plasma*, *PRP*, *hydrodissection*, *suprascapular nerve*, *axillary nerve*, *ultrasound-guided injection*, *shear-wave elastography*, and *wearable sensor*. The search window was extended to April 2026 to capture recently published and early-access trials and meta-analyses, and is consistent with all cited 2026 sources.

**Inclusion criteria.** English-language randomized controlled trials (RCTs), prospective comparative cohort studies, systematic reviews, and meta-analyses addressing AC and the interventions above were prioritized. Selected mechanistic, anatomical, and imaging-validation references were retained when they clarified clinical reasoning or supported the reliability of an outcome tool.

**Exclusion criteria.** We excluded non-shoulder studies (except where a mechanistic or measurement-property extrapolation was explicitly flagged as such), conference abstracts without full text, and, for efficacy claims, single case reports (case-level evidence is cited only where AC-specific comparative data are absent and is explicitly identified as such in the text).

**Selection and quality appraisal.** The initial search yielded approximately 1200 records. After the removal of duplicates and title/abstract screening for AC relevance, comparative or mechanistic content, and ultrasound-guided technique, 218 records were retained for full-text review. Of these, 55 publications are cited in the present review. Because this is a narrative synthesis, we did not perform meta-analytic pooling. However, to give readers an explicit sense of the strength of the underlying evidence, we appraised the risk of bias of the pivotal AC-specific RCTs using the domains of the revised Cochrane Risk of Bias tool (RoB 2) [19]; the resulting judgments, together with study design characteristics (single- versus multicenter; blinded versus open-label; a priori power calculation), are summarized in Table 2. We acknowledge that informal source selection introduces selection bias and that this review cannot substitute for a formal systematic review or a formal GRADE assessment.

## 3. Pathophysiology and Clinical Phases of Adhesive Capsulitis

### 3.1. Inflammatory–Fibrotic Cascade

Current biological models describe AC as a sequential transition from synovial inflammation to capsular fibrosis [20,21]. In the early freezing phase, the synovium and rotator interval show dense neovascularization and infiltration by mast cells, T lymphocytes, and macrophages, alongside upregulation of pro-inflammatory cytokines (IL-1β, IL-6, TNF-α) and pro-fibrotic mediators (TGF-β, PDGF, CTGF) [20,21]. As the disease progresses, fibroblast-to-myofibroblast transition drives excessive deposition of type I and III collagen, contracture of the coracohumeral and inferior glenohumeral ligaments, and obliteration of the axillary recess—the structural correlate of the global capsular-pattern restriction observed clinically [20,21]. Once mature fibrosis has formed, mechanical stretching alone progressively loses effectiveness, which is the biological argument for intervening earlier, before adhesion becomes structural rather than reversible.

### 3.2. Neurogenic Component

The glenohumeral joint is innervated principally by the suprascapular nerve, with contributions from the axillary and subscapular nerves. In AC, these nerves may undergo neurogenic sensitization: increased nerve growth factor expression in the inflamed capsule, perineural fibrosis at fascial constriction sites (notably the suprascapular notch and quadrilateral space), and central sensitization can amplify nociceptive output [22]. This mechanism helps explain why severe pain may persist even after capsular extensibility improves, and it provides the rationale for treating AC with perineural hydrodissection in addition to—not instead of—capsular pharmacotherapy.

### 3.3. Clinical Staging and Therapeutic Implications

Clinically, AC is divided into four phases—painful (pre-freezing), freezing, frozen, and thawing—though in practice they overlap. Each phase carries different therapeutic priorities. The freezing phase responds best to inflammation-modulating, pain-controlling interventions that allow rehabilitation to begin. The frozen and thawing phases require capsular distension and graded motion. Recognizing the dominant pathological process in each stage is the conceptual foundation of the phase-specific framework proposed in Section 9.

## 4. The Corticosteroid Paradigm: Benefits, Limits, and Why Alternatives Are Needed

### 4.1. Short-Term Efficacy and Mechanistic Basis

Intra-articular corticosteroids have been the cornerstone of pharmacological management in AC for decades. It acts primarily by suppressing synovial inflammation, dampening pro-inflammatory cytokine cascades, and reducing nociceptive sensitization. Multiple randomized trials and the *JAMA Network Open* network meta-analysis confirm that corticosteroids produce faster pain reduction in the first 4–6 weeks than placebos, oral NSAIDs, or physiotherapy alone [9,23]. In the freezing phase, corticosteroids offer the most rapid analgesic onset among available options, and this capacity to permit earlier engagement in rehabilitation remains clinically useful.

### 4.2. Limitations: Durability, Diabetic Risk, Tissue Toxicity

Three limitations consistently temper enthusiasm for corticosteroids as a stand-alone strategy. First, durability is short: beyond 12 weeks, between-group differences against placebos, HA, or rehabilitation tend to attenuate or disappear, and recurrence within six months is common [24,25]. Second, diabetic patients face disproportionate risk: a single intra-articular triamcinolone injection produces measurable hyperglycemia for 24–72 h, with mean glucose elevations of approximately 30–80 mg/dL and prolonged elevations in those with poor baseline control [26]; repeated injections cumulatively worsen glycemic control and may interact with comorbid microvascular disease. Third, repeated administration carries tissue costs: preclinical and clinical data link multiple intra-articular corticosteroid exposures to chondrocyte apoptosis, glycosaminoglycan loss, and progression of cartilage degeneration, while peri-tendinous corticosteroids weaken collagen architecture. It should be noted that the most-cited chondrotoxicity signal derives from a knee osteoarthritis trial [27] rather than from glenohumeral data; extrapolation to the shoulder is therefore mechanistically plausible but not directly demonstrated. In middle-aged patients with concurrent rotator cuff pathology, repeated steroid use may accelerate tendon failure.

### 4.3. The Functional Plateau and the Risk of Chronic Adhesions

Even when corticosteroids produce early pain relief, functional recovery often plateaus before patients regain the multi-planar mobility required for everyday tasks—overhead dressing, dorsal-reach hygiene, lifting at work. The mismatch is mechanistic: corticosteroids suppress inflammation and pain but does not act directly on capsular fibrosis or perineural sensitization. The clinical consequence is that the disease can shift, largely silently, from a reversible inflammatory state to a structurally adhesive one. A reasonable response is to introduce non-steroidal alternatives—regenerative injectates for the fibrotic capsule, hydrodissection for the sensitized perineural environment—and to introduce them before the corticosteroid response has plateaued rather than after (Table 1).

**Table 1:** Comparison of management paradigms for adhesive capsulitis: prevailing recommendations versus the proposed proactive non-steroidal-first framework. An evidence-level annotation is provided for each domain to indicate the strength of support (RCT + MA = supported by randomized trials and at least one meta-analysis/systematic review; RCT = at least one randomized trial; EXTRAP = evidence extrapolated from adjacent pathology; EXP = clinical experience/framework-level).

Domain	Prevailing Corticosteroid-First Paradigm [8,9,10]	Proposed Proactive Non-Steroidal-First Framework (This Review)	Evidence Level for Proposed Position
First-line freezing-phase agent	Intra-articular corticosteroid	Ultrasound-guided HA, dextrose, or PRP (agent by subgroup)	HA, PRP: RCT + MA [11,12,13,28,29]; dextrose: EXTRAP [30,31]
Primary goal	Rapid short-term pain relief	Rapid pain control plus durable capsular modulation	RCT + MA [11,12,13,28]
Role of perineural hydrodissection	Reserved for refractory pain	SSN hydrodissection as freezing-phase adjunct when pain limits rehabilitation	SSN: RCT [15,32]; axillary/subscapular: EXP/case-level [33]
Diabetic patients	Corticosteroids with glycemic caution	Non-steroidal injectate preferred; D5W hydrodissection for analgesia	RCT + MA [12,13,28]; EXP [26]
Rehabilitation	Central	Central (principal driver of functional recovery)	RCT [34,35]
Objective monitoring	VAS/SPADI/ROM	Add SWE and wearable IMU where available	Emerging/validation-stage [16,17,36,37,38]
Overall status	Guideline-anchored standard	Clinically defensible framework, not yet prospectively validated	EXP/framework

## 5. Ultrasound-Guided Regenerative Injectates

**Figure 2:**
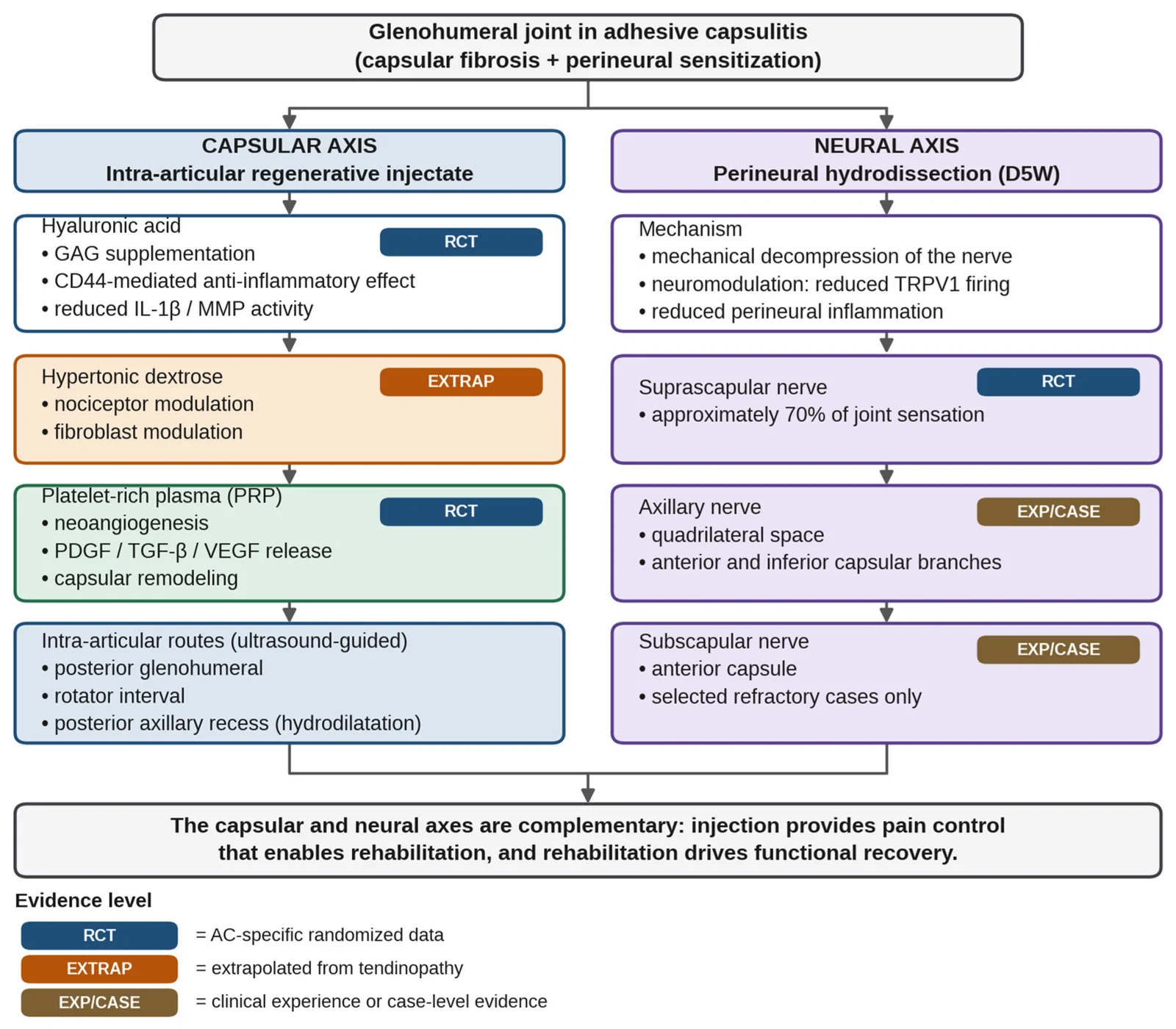
Schematic illustration of the two therapeutic axes in adhesive capsulitis and their mechanisms and anatomical targets. The capsular axis (left) depicts intra-articular regenerative injectates—hyaluronic acid (glycosaminoglycan supplementation, CD44-mediated anti-inflammatory effect), hypertonic dextrose (nociceptor and fibroblast modulation), and PRP (growth-factor-driven neoangiogenesis and capsular remodeling)—delivered via posterior glenohumeral, rotator interval, and axillary recess routes. The neural axis (right) depicts perineural hydrodissection of the suprascapular, axillary, and subscapular nerves (mechanical decompression plus TRPV1-related neuromodulation). Arrows indicate the branching relationship between the disease state, each therapeutic axis, and its anatomical targets; they do not denote a temporal sequence of administration. Evidence strength is annotated per target. AC, adhesive capsulitis; D5W, 5% dextrose in water; GAG, glycosaminoglycan; PRP, platelet-rich plasma.

The mechanisms of action and anatomical targets of the regenerative injectates (Section 5) and perineural hydrodissection strategies (Section 6) are summarized schematically in Figure 2, which distinguishes the capsular axis from the neural axis of treatment.

### 5.1. Hyaluronic Acid: Viscosupplementation and Capsular Modulation

HA is the most extensively studied non-steroidal injectate in AC. Beyond its viscoelastic lubricating role, intra-articular HA has anti-inflammatory effects via CD44 receptor binding, suppresses nociceptive signaling, downregulates IL-1β-induced metalloproteinase activity, and modulates TGF-β-driven fibroblast proliferation in capsular tissue [39,40]. These mechanisms translate into pain reduction and functional gain in clinical practice, particularly when delivery is ultrasound-guided to confirm intra-articular placement. Multiple randomized trials and meta-analyses report that HA matches corticosteroids in pain and SPADI outcomes at 3–6 months and may offer greater durability beyond 12 weeks [28,29].

HA also pairs well with rehabilitation because it lowers the pain barrier to passive capsular stretch rather than substituting for it. In a single-center, parallel-group randomized trial of 46 adults with frozen-phase AC (HA group n = 21; supervised-rehabilitation group n = 25), three weekly ultrasound-guided intra-articular non-cross-linked HA injections produced clinically meaningful improvement in pain, function, and shoulder mobility that was statistically equivalent to six weeks of supervised rehabilitation across 26-week follow-up [34]. Total SPADI decreased from 43.03 ± 17.65 to 16.14 ± 12.77 in the HA group (a 62.5% reduction) and from 51.97 ± 18.35 to 23.21 ± 21.83 in the rehabilitation group (a 55.3% reduction); both within-group changes were significant (*p* < 0.01), and between-group differences were not statistically significant at any timepoint (*p* > 0.05). Active flexion improved from 111.19° ± 14.31 to 128.00° ± 12.29 (HA) and from 103.80° ± 26.59 to 128.16° ± 21.23 (rehabilitation), and active abduction improved from 94.05° ± 14.72 to 119.75° ± 17.51 (HA) and from 85.00° ± 30.24 to 115.79° ± 30.01 (rehabilitation), with no consistent between-group advantage. The trial was powered a priori to detect a 10-point between-group SPADI difference (assumed SD 12) at 80% power and two-tailed α = 0.05, requiring at least 21 participants per group, and no adverse events were reported [34]. Two interpretive caveats are important. First, the comparator was supervised rehabilitation rather than corticosteroids, so the trial establishes therapeutic equivalence to rehabilitation, not superiority over steroids. Second, it is a single-center study; its point estimates should be regarded as preliminary pending multicenter replication. Notably, this trial was disclosed as “in press” in the originally submitted version of this review and is now formally published, so its full methods and results are available for independent scrutiny. For patients with diabetes mellitus, or those who decline corticosteroids, HA is a reasonable first choice.

### 5.2. Hypertonic Dextrose Prolotherapy

**Figure 3:**
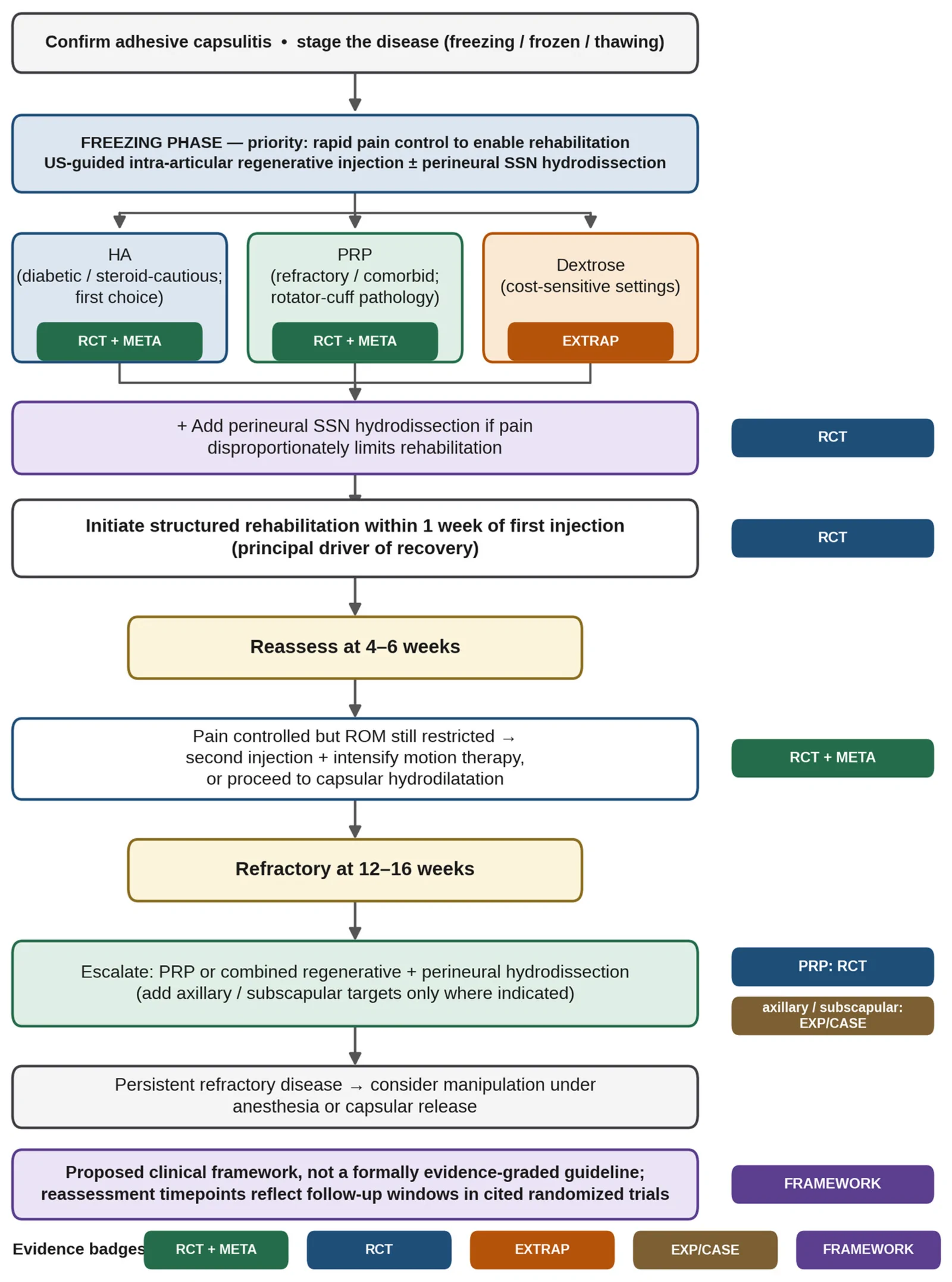
Phase-specific, ultrasound-guided treatment framework for adhesive capsulitis: a proposed clinical algorithm. This algorithm integrates current evidence on regenerative injectates [11,12,13,28,29] and perineural hydrodissection [14,15,32,33,42] with the authors’ clinical experience at a tertiary rehabilitation center. It is a conceptual framework to support individualized decision-making, not a formally evidence-graded guideline. Evidence-level badges mark each node: RCT + META = multiple randomized trials and at least one meta-analysis/systematic review; RCT = at least one randomized trial; EXTRAP = evidence extrapolated from adjacent pathology (**applied to the hypertonic-dextrose node**, whose comparative RCTs concern rotator cuff tendinopathy rather than AC); EXP/CASE = clinical experience or case-level evidence (**applied to the axillary and subscapular hydrodissection nodes**); FRAMEWORK = clinical synthesis. Reassessment timepoints (4–6 and 12–16 weeks) reflect follow-up windows in cited RCTs [15,34,35]. AC, adhesive capsulitis; HA, hyaluronic acid; PRP, platelet-rich plasma; ROM, range of motion; SSN, suprascapular nerve.

Prolotherapy with hypertonic dextrose (typically 12.5–25%), injected intra-articularly or peri-capsularly, induces a controlled, low-grade inflammatory response. The historical rationale was tissue augmentation through fibroblast proliferation and collagen reorganization; in AC, the more credible mechanisms are modulation of nociceptive afferents and capsular fibroblast behavior, with downstream effects on pain and capsular extensibility [41]. Recent randomized comparisons report that intra-articular dextrose produces pain and disability improvements comparable to, or modestly better than, those of corticosteroids at 12–24 weeks. **A critical caveat must accompany this claim at the point of recommendation:** the published comparative evidence for dextrose derives largely from related shoulder pathologies—particularly rotator cuff tendinopathy—rather than from adhesive-capsulitis-specific cohorts [30,31], and AC-specific randomized data remain limited. Dextrose should therefore be regarded as a mechanistically plausible, evidence-supported option in adjacent shoulder disorders whose AC-specific efficacy is still being established, and this distinction is flagged explicitly in the step-up framework (Section 9 and Figure 3). The systemic glycemic load from typical injection volumes is negligible, conferring an advantage over corticosteroids for diabetic patients while AC-specific data accumulate.

What makes prolotherapy clinically attractive is its safety, low cost, and accessibility: no autologous blood processing, no proprietary preparations, and no recognized chondrotoxicity with repeated administration. These features make dextrose particularly suited to resource-limited settings, to patients who decline steroids, and to those with multiple comorbidities. The principal obstacles are the heterogeneity of injection volumes, concentrations, and protocols across trials, which slows formal standardization, and the fact that much of the supporting rationale is extrapolated from adjacent shoulder disorders. Balanced critical appraisals of orthobiologic injectates emphasize precisely this gap between biological rationale and disease-specific clinical evidence [43].

### 5.3. Platelet-Rich Plasma: Durable Biological Augmentation

PRP delivers concentrated platelet-derived growth factors—PDGF, TGF-β, VEGF, EGF, IGF-1—and a smaller cytokine load to the injection site. In AC, the biological rationale is twofold: modulation of capsular fibroblast behavior and enhancement of capsular angiogenesis, both of which may shift the capsule toward remodeling rather than scar maturation. Comparative data have accumulated to the point that PRP need not be treated only as a refractory rescue. In a single-center randomized trial of 202 frozen-shoulder patients, intra-articular PRP produced greater improvements in shoulder ROM and UCLA shoulder score than methylprednisolone at 12 weeks, with mean abduction reaching 147° in the PRP arm versus 129° in the corticosteroid arm; the source report did not provide confidence intervals or standardized effect sizes for these outcomes [11]. A subsequent single-center randomized study of 68 patients followed for 24 weeks found that intra-articular PRP yielded superior VAS, QuickDASH, and SPADI outcomes compared with corticosteroids, with comparable safety [12]. A 2026 systematic review and meta-analysis reported a consistent pattern: at 1 month PRP and corticosteroids were roughly equivalent, whereas at 3 and 6 months PRP was superior for pain, function, and ROM, with similarly low complication rates [13].

Two limitations should be weighed against these findings. First, the two pivotal RCTs [11,12] are single-center and open-label. Absence of participant and assessor blinding introduces potential performance and detection bias, and for a subjectively rated novel intervention this bias would be expected to favor the PRP arm; effect estimates are therefore likely to represent an upper bound and may attenuate in blinded, multicenter replication (Table 2). Second, PRP preparations are heterogeneous—leukocyte-rich versus leukocyte-poor, variable platelet concentration, and differing activation methods—and the cited trials seldom report these parameters in a standardized way. Adoption of a formal reporting scheme such as the PAW (Platelets–Activation–White cells) classification [44] would improve comparability across studies. With these caveats, PRP is a defensible primary option for diabetic patients, for patients who decline steroids, and for those with concomitant rotator cuff pathology in whom regenerative augmentation is desirable; the barriers of cost, preparation variability, and uneven reimbursement remain real.

**Table 2:** Randomized controlled trials and meta-analyses cited as principal evidence for ultrasound-guided non-steroidal injection paradigms in adhesive capsulitis, with design characteristics and risk-of-bias appraisal using the domains of the revised Cochrane Risk of Bias tool (RoB 2) [19]. PRP, platelet-rich plasma; HA, hyaluronic acid; SSN, suprascapular nerve; RCT, randomized controlled trial; SR/MA, systematic review/meta-analysis; NR, not reported; N/A, not applicable (trial-level risk-of-bias domains are not applicable to systematic reviews or meta-analyses).

Study (Ref)	Design/n	Comparison	Key Outcome(s)	A Priori Power?	Blinding/Centers	RoB 2 Overall Judgment
Shahzad, 2021 [11]	RCT, n = 202	PRP vs. corticosteroid	ROM, UCLA at 12 wk (abduction 147° vs. 129°); CIs/effect sizes NR	NR	Open-label, single-center	Some concerns—high (unblinded; bias likely favors PRP)
Somisetty, 2023 [12]	RCT, n = 68	PRP vs. corticosteroid	VAS, QuickDASH, SPADI to 24 wk (PRP superior)	NR	Open-label, single-center	Some concerns—high (unblinded; bias likely favors PRP)
Xu, 2026 [13]	SR/MA of RCTs	PRP vs. corticosteroid	Pain, function, ROM at 1/3/6 mo (PRP superior at 3–6 mo)	N/A	Includes small single-center RCTs	Moderate confidence; limited by primary-study bias
Wu, 2017 [15]	RCT	US-guided SSN block + rehab vs. rehab	Pain, ROM sustained to 12 wk	NR	Single-center	Some concerns
Chang, 2026 [34]	RCT, n = 46	US-guided HA vs. supervised rehabilitation	SPADI, ROM to 26 wk (equivalent; SPADI −62.5% vs. −55.3%)	Yes (10-pt SPADI, SD 12, 80% power)	Assessor-context single-center	Low—some concerns
Wu, 2024 [35]	RCT	US-guided HA hydrodilatation + PT vs. PT	SPADI, ROM, PROMs; gains by 3 mo, held to 6 mo	NR	Single-center	Some concerns

### 5.4. Comparative Efficacy and Durability vs. Corticosteroids

Across head-to-head comparisons a consistent pattern emerges: corticosteroids act faster but for a shorter duration, whereas non-steroidal injectates act more slowly but more durably. At 4–8 weeks corticosteroids usually produce the largest pain reduction; by 12–24 weeks HA, PRP, and dextrose typically match or surpass it; and beyond 24 weeks the durability advantage of non-steroidal options is most apparent [9,11,12,13,28,30]. Direct comparisons among the three regenerative agents are scarce, and the available data do not identify a single dominant agent. Agent choice should therefore follow patient-specific factors—disease stage, glycemic status, prior injection history, cost and reimbursement, and concomitant pathology—as discussed in Section 8. On balance, the evidence suggests that a corticosteroid-first default may not be optimal for patients whose long-term autonomy and avoidance of recurrent flares are the priority, although this inference rests on medium-term data from predominantly single-center trials and awaits confirmation in head-to-head studies.

## 6. Ultrasound-Guided Perineural Hydrodissection

### 6.1. Mechanism: Mechanical Decompression Plus Neuromodulation

Hydrodissection is the ultrasound-guided injection of fluid—usually normal saline, 5% dextrose in water (D5W), or low-concentration anesthetic—into the perineural space, separating the nerve from surrounding fascia and inflamed connective tissue. The proposed mechanism is dual: mechanical decompression of compressed or tethered segments, and neuromodulation through local effects on perineural inflammation and aberrant ion-channel activity, particularly TRPV1 [14]. D5W has emerged as a preferred fluid because it avoids corticosteroid-associated risks, eliminates the neurotoxicity concerns of repeated local anesthetic exposure, and appears to have analgesic effects beyond pure mechanical separation [45]. In AC, hydrodissection targets perineural sensitization—something intra-articular pharmacotherapy cannot address—and is most relevant for patients whose pain is out of proportion to their stiffness and who therefore cannot tolerate the stretching they need. Importantly, much of this mechanistic rationale derives from the broader perineural hydrodissection literature, including entrapment neuropathies such as carpal tunnel syndrome [14,45]; AC-specific randomized evidence for perineural hydrodissection remains limited, and parts of the rationale presented here are extrapolated from related conditions.

### 6.2. Suprascapular Nerve

The suprascapular nerve (SSN) supplies up to 70% of glenohumeral joint sensation and is the highest-yield perineural target in AC, with the strongest supporting evidence among the perineural approaches discussed here. An early randomized trial showed that a single SSN block produced faster and more complete pain resolution and ROM restoration than a series of intra-articular injections in primary-care AC [32]. More recent ultrasound-guided trials confirm that perineural intervention at the SSN—with anesthetic, anesthetic plus steroid, or D5W—shortens the pain trajectory and improves rehabilitation tolerance compared with rehabilitation alone [15]. The technique is reliable: the transducer is placed in the supraspinous fossa, the needle directed lateral-to-medial toward the floor of the fossa, and 5–10 mL of D5W or normal saline deposited around the nerve, with the trapezius and supraspinatus superficial and the suprascapular notch medial. Pain at rest and during motion typically falls within minutes. In our experience, SSN hydrodissection is particularly valuable as a bridge intervention before initiating aggressive stretching.

### 6.3. Axillary Nerve

The axillary nerve traverses the quadrilateral space (bounded by teres minor, teres major, the long head of triceps, and the humeral neck) and innervates the inferior glenohumeral capsule and posterior shoulder. In AC, axillary nerve sensitization may contribute to posterior and inferior shoulder pain and to pain on abduction. The nerve is approached posteriorly with the patient prone or in lateral decubitus, with the target identified deep to the deltoid and adjacent to the posterior circumflex humeral artery. **The evidence level here differs materially from that for the SSN:** AC-specific support for axillary hydrodissection rests on case-level evidence and clinical experience rather than randomized trials [33]. Axillary hydrodissection should therefore be regarded as a reasonable adjunct in selected patients with prominent posterior pain or restricted abduction, offered with explicit acknowledgement of its limited evidence base, and this qualification is carried through to the step-up framework in Section 9.

### 6.4. Subscapular Nerve

The upper and lower subscapular nerves innervate the subscapularis and contribute, with the SSN, to anterior glenohumeral capsular sensation. In selected AC patients with prominent anterior pain or rotator interval involvement, hydrodissection of the subscapular nerve in the axillary fossa may supplement SSN/axillary blockade. This target is anatomically more variable, is supported by clinical experience rather than controlled AC-specific data, and is best reserved for refractory cases or for clinicians experienced in anterior shoulder sonography. As with the axillary target, we flag this as an experience-level recommendation.

### 6.5. Combined Multi-Nerve Approaches

Some clinicians combine SSN and axillary (and selectively subscapular) hydrodissection in the same session for broader analgesic coverage. Randomized data on combined protocols remain limited, and a small randomized comparison of suprascapular nerve block with two hydrodilatation targets found no clear superiority of any single approach; target selection therefore rests on the pain phenotype rather than on demonstrated comparative advantage [42]. The combined approach is best regarded as an adjunct to—not a substitute for—intra-articular injectate therapy in the freezing phase: it represents the neural axis of treatment, complementary to the capsular axis.

## 7. Anatomy and Injection Approaches

Ultrasound guidance is the unifying principle of contemporary AC injection: it confirms intra-articular or perineural placement, helps avoid critical structures, and lets the operator observe fluid distribution in real time. Systematic reviews of injection accuracy consistently show that image guidance improves needle-placement accuracy over the palpation-guided technique, an advantage that is most pronounced for deep or technically demanding targets [46]. This benefit is, however, operator-dependent (Section 8.4). The anatomical approach varies by target.

### 7.1. Intra-Articular Routes

**Posterior glenohumeral approach.** This is the most widely used technique. The patient lies in lateral decubitus or prone, the transducer is placed transversely over the posterior glenohumeral joint to identify the posterior glenoid, humeral head, and infraspinatus, and a 22-gauge needle is advanced in-plane, lateral-to-medial, into the joint cavity. Intra-articular placement is confirmed by visualizing fluid flow across the joint surface. This route is reliable in most patients and is the one used in the majority of clinical trials of HA, prolotherapy, PRP, and corticosteroids [34].

**Rotator interval approach.** With the transducer placed transversely over the anterior shoulder, the rotator interval—bordered by supraspinatus, subscapularis, the biceps long head, and the coracoid process—is identified. The needle is advanced from the lateral side, with the tip placed in the rotator interval beneath the coracohumeral ligament. This approach is favored for delivering HA or anti-fibrotic agents directly to the rotator interval, the region most strongly implicated in early capsular fibrosis [47].

**Posterior axillary recess approach.** The axillary recess is the inferior pouch of the joint capsule, classically obliterated in advanced AC. With the patient seated and the arm forward-flexed, the transducer is placed inferiorly in the axilla to image the recess. Direct injection here is technically demanding but provides an efficient route for capsular distension (high-volume hydrodilatation), increasingly used to mechanically disrupt capsular adhesions [48]. Systematic-review evidence indicates that hydrodilatation produces at least transient improvements in disability and passive external rotation relative to corticosteroids alone, although the durability and clinical magnitude of this benefit remain debated [49].

### 7.2. Perineural Targets and Sonographic Landmarks

**SSN.** Supraspinous fossa, between trapezius and supraspinatus, needle directed toward the floor; the suprascapular vessels accompany the nerve. **Axillary nerve.** The quadrilateral space is approached posteriorly, with the nerve identified deep to the deltoid and adjacent to the posterior circumflex humeral artery. **Subscapular nerves.** Anterior axillary fossa, deep to pectoralis major and adjacent to the subscapularis muscle belly; this approach demands experience and careful identification of neurovascular structures.

### 7.3. Practical Pearls

In the freezing phase the joint capacity is markedly reduced (5–10 mL versus 15–30 mL in healthy shoulders), so resistance during injection should be expected; forceful injection risks extra-articular extravasation. For perineural hydrodissection, intraneural injection must be avoided by maintaining a clear sonographic view of the needle tip outside the nerve epineurium, and a low-volume test injection to confirm peri- (not intra-) neural spread is good discipline. Across all approaches, sterility, transducer–needle alignment, and Doppler imaging to identify accompanying vessels are essential.

## 8. Patient Subgroup Considerations, Safety, and Practical Limitations

### 8.1. Diabetes Mellitus and Metabolic Comorbidity

Diabetic patients are the most important subgroup in whom corticosteroids alone is suboptimal. Post-injection hyperglycemia, more aggressive capsular fibrosis, and higher recurrence together make non-steroidal injectates—HA, dextrose prolotherapy, and PRP—the preferred first-line pharmacological options [12,13,26,28,30]. For diabetic patients with severe pain limiting rehabilitation, perineural hydrodissection with D5W provides analgesia without glycemic perturbation and is a reasonable adjunct to intra-articular regenerative injectate.

### 8.2. Refractory and Long-Standing Disease

Patients with refractory AC—operationally, persistent disability beyond 6 months despite at least one injection cycle and adequate rehabilitation—warrant escalation: PRP, high-volume capsular hydrodilatation, or combined regenerative-plus-hydrodissection strategies [13,48,49]. Mechanical capsular distension (often ≥20 mL) directly addresses the fibrotic restriction that pharmacological approaches alone cannot release.

### 8.3. Contraindications to Repeated Steroid Use

Poorly controlled diabetes, prior multiple steroid injections (typically ≥3 to the same shoulder), concomitant rotator cuff tendinopathy or partial-thickness tear, and osteoporosis-related fragility are further indications for non-steroidal alternatives. In these subgroups, the durability and tissue-sparing profile of HA, PRP, and dextrose prolotherapy align with long-term clinical objectives.

### 8.4. Safety Profile, Adverse Events, and Operator Dependence

Any recommendations to broaden the use of non-steroidal injections must be balanced against a clear account of safety and practical limitations. In the cited AC trials, the non-steroidal injectates were generally well tolerated: the HA-versus-rehabilitation trial reported no adverse events [34], and the PRP trials reported complication rates comparable to corticosteroids [11,12,13]. Nonetheless, several injectate- and procedure-specific considerations warrant attention. Intra-articular HA and PRP may cause transient post-injection pain or a self-limited inflammatory flare, and PRP in particular is associated with a short-lived post-injection soreness attributable to its pro-inflammatory action. Hypertonic dextrose can cause an osmotic injection-site ache. General injection contraindications—overlying skin or joint infection, uncontrolled coagulopathy or therapeutic anticoagulation, and known hypersensitivity to a specific agent—apply to all of these injectates.

Perineural hydrodissection carries its own procedure-specific risks: inadvertent intraneural injection (mitigated by continuous visualization of the needle tip outside the epineurium and a low-volume test injection), vascular puncture of accompanying vessels (mitigated by pre-procedural Doppler), and, rarely, transient motor block. Two structural limitations further temper enthusiasm. First, all of these techniques are **operator-dependent**: the accuracy advantage of ultrasound guidance is realized only with adequate training and a sufficient procedural learning curve, and image-guided accuracy varies with operator experience and target depth [46]. Second, the absence of standardized preparation and dosing protocols—particularly for PRP [44] and dextrose—limits reproducibility across centers and remains a barrier to wider, evidence-based adoption. These considerations argue for careful patient selection, adequate operator training, and transparent protocol reporting rather than indiscriminate use.

## 9. A Phase-Specific Treatment Framework

### 9.1. Freezing Phase: Pain Control and Capsular Release

The freezing phase is dominated by inflammation-driven pain that is disproportionate to ROM loss, so the therapeutic priority is rapid pain control to allow rehabilitation to begin without exacerbation. A single intra-articular injection (HA, dextrose, or PRP), with or without adjunctive perineural SSN hydrodissection, is usually sufficient to lower the pain barrier. Agent selection should reflect the evidence hierarchy discussed above: HA and PRP are supported by AC-specific randomized data, whereas dextrose is supported chiefly by evidence extrapolated from rotator cuff tendinopathy, and SSN hydrodissection carries stronger AC-specific support than axillary or subscapular hydrodissection. Corticosteroids remain an option for non-diabetic patients who require maximally rapid analgesia; given durability and safety considerations, however, non-steroidal alternatives are increasingly favored as first-line in the subgroups identified in Section 8.

### 9.2. Frozen-to-Thawing Transition: Structured Rehabilitation

As pain subsides, the dominant pathology shifts from inflammation to mechanical restriction, and the therapeutic focus shifts accordingly—toward capsular extensibility and active motion restoration. Structured rehabilitation—combining progressive passive and active-assisted stretching across all planes, periscapular strengthening, and neuromuscular retraining—becomes the principal driver of recovery. A second or third HA or PRP injection at 2–6-week intervals can sustain capsular lubrication and pain control during this rehabilitation-intensive period [28]. It bears emphasis that the injection is an enabling adjunct; rehabilitation performs the functional work.

### 9.3. The 3–6-Month Recovery Window: A Hypothesis Supported by Preliminary Evidence

We propose—as a hypothesis rather than an established outcome—that timely ultrasound-guided injection paired with stage-appropriate rehabilitation may compress the symptomatic course of AC from years to approximately three to six months in appropriately selected patients. The supporting evidence is suggestive but limited and heterogeneous. Wu and colleagues showed that a single ultrasound-guided SSN block accelerated pain and ROM recovery when combined with conventional rehabilitation, with benefits sustained at 12 weeks [15]. A subsequent randomized trial from the same group demonstrated that ultrasound-guided hydrodilatation with HA plus physical therapy outperformed physical therapy alone on SPADI, ROM, and patient-reported outcomes, with most gains realized within the first three months and maintained at six months [35]. A complementary parallel-group randomized trial in frozen-phase AC showed that ultrasound-guided intra-articular HA produced clinically meaningful functional recovery equivalent to supervised rehabilitation across a 26-week follow-up [34]. The PRP trials cited above report comparable durable functional gains at 12–24 weeks [11,12,13].

These trials differ substantially in intervention (SSN block; HA hydrodilatation; HA versus rehabilitation; PRP versus steroids), comparator, and outcome timing, so the “3–6 months” figure should be read as a plausible target derived from convergent but non-identical trial conditions rather than as a validated characteristic of a single unified protocol. What the trials share is a common structure: an ultrasound-guided non-steroidal injection acts as a pain-control catalyst that opens a therapeutic window for rehabilitation, which then drives functional recovery. The qualifier matters in clinical settings—injection alone is not a substitute for rehabilitation, and rehabilitation alone is often slowed by uncontrolled pain. The sequence we favor is an ultrasound-guided non-steroidal injection in the freezing phase, followed without delay by graded motion therapy, progressing from gentle passive mobilization to active multi-planar functional training as pain subsides. Confirmation of the recovery window awaits a prospective trial of the integrated paradigm.

### 9.4. A Pragmatic Step-Up Framework

The step-up framework summarized in Figure 3 is a proposed clinical algorithm rather than an evidence-graded guideline. It integrates the cited randomized and meta-analytic evidence for each intervention node with the authors’ clinical experience in a tertiary rehabilitation center and is intended to support individualized decision-making. Evidence levels for each node are indicated within the figure, and nodes supported chiefly by extrapolated or case-level evidence (dextrose prolotherapy; axillary and subscapular hydrodissection) are flagged as such so that the algorithm does not overstate AC-specific certainty.

A step-up framework for the typical AC patient can be summarized as follows. (i) In the freezing phase, deliver an ultrasound-guided intra-articular regenerative injection—HA preferred for diabetic and steroid-cautious patients (AC-specific RCT support), PRP for refractory or comorbid cases (AC-specific RCT support), dextrose for cost-sensitive settings (evidence largely extrapolated from tendinopathy)—and add perineural SSN hydrodissection (the best-supported perineural target) if pain disproportionately limits rehabilitation. (ii) Initiate structured rehabilitation within one week of the first injection. (iii) Reassess at 4–6 weeks; if pain is controlled but ROM remains restricted, consider a second injection and intensify motion therapy, or proceed to capsular hydrodilatation. (iv) For refractory disease at 12–16 weeks, escalate to PRP or combined regenerative-plus-hydrodissection strategies (adding axillary/subscapular targets only where clinically indicated and with acknowledgement of their limited evidence base) before considering manipulation under anesthesia or capsular release. This sequence operationalizes the proactive framework and is, in our practice, the pathway most consistent with the hypothesized 3–6-month recovery window; it has not, however, been validated prospectively as a single protocol.

## 10. Mechanistic Bridges Across Disease Stages

### 10.1. Glycosaminoglycan Supplementation

Within the joint cavity, exogenous HA restores synovial viscoelasticity, replenishes the depleted glycosaminoglycan pool of inflamed capsular tissue, and binds CD44 receptors on fibroblasts and synoviocytes to suppress IL-1β, IL-6, and matrix metalloproteinase activity [39,40]. This mechanism is most relevant in the freezing and early frozen phases, when synovial inflammation and capsular hydration deficits dominate.

### 10.2. Neoangiogenesis and Tissue Remodeling

PRP, and to a lesser extent prolotherapy, stimulate controlled neoangiogenesis and modulate fibroblast-to-myofibroblast dynamics. In the frozen and thawing phases—when fibrosis predominates—these mechanisms may favor capsular remodeling rather than maturation of inert scars [12,13]. The choice of regenerative agent thus aligns with the disease stage: HA for inflammation-dominant phases, and PRP and prolotherapy for fibrosis-dominant phases. We note that this stage-agent alignment is a mechanistically motivated framework rather than a directly trial-tested prescription.

### 10.3. Modulation of Neural Sensitization

Perineural hydrodissection, especially with D5W, is thought to reduce aberrant TRPV1-mediated neural firing, decompress tethered nerve segments, and disrupt the local inflammatory milieu of sensitized nerves [14,45]. This mechanism is relevant across all phases but is most clinically valuable when pain disproportionately limits motion therapy. Combining intra-articular injectate with perineural hydrodissection therefore addresses the capsular and neural dimensions of AC simultaneously rather than only one.

## 11. Objective Monitoring of Recovery: Closing the Feedback Loop

Conventional outcome assessment in AC—VAS for pain, SPADI or DASH for function, goniometric ROM—captures only part of recovery and is vulnerable to expectation bias and rater variability. Notably, even these standard instruments have quantifiable measurement properties that should inform their interpretation: the SPADI demonstrates good test–retest reliability in AC populations (reported intraclass correlation coefficient ~0.89 and Cronbach’s α ~0.94) [50], and anchor- and distribution-based analyses place the SPADI total-score minimal clinically important difference (MCID) in the region of 17 points (95% CI approximately 13.6–20.4) in shoulder surgical cohorts [51]. These values frame the ~60% SPADI reductions reported in Section 5.1 as changes that comfortably exceed measurement error and the MCID. Three emerging tools may extend assessment beyond these instruments, though their validation specifically in AC remains at an early stage.

### 11.1. High-Resolution Ultrasound

Modern high-frequency probes (≥18 MHz) allow direct visualization of the coracohumeral ligament, axillary recess, and rotator interval. Quantitative ultrasound markers—coracohumeral ligament thickness, axillary recess capacity, capsular vascularity on Doppler—correlate with disease severity and symptom duration [52], offering a more biologically meaningful index of capsular remodeling than pain scores alone. We note that the reported probe frequency is a minimum specification; specific transducer model, manufacturer, and machine settings vary across the cited studies and should be reported in primary trials to support reproducibility.

### 11.2. Shear-Wave Elastography

Shear-wave elastography (SWE) quantifies tissue stiffness in vivo and is an increasingly useful tool for objectively characterizing capsular fibrosis in AC. Several studies report that SWE- and strain-derived stiffness values of the coracohumeral and inferior glenohumeral ligaments and the peri-articular tendons distinguish AC from healthy shoulders and change measurably with treatment [16,36,53]. Reported reliability for shoulder SWE is moderate to good but is operator- and system-dependent, so absolute stiffness thresholds are not yet transferable across machines. With standardization, SWE may serve as a non-invasive surrogate of capsular extensibility, complementing pain and ROM endpoints and providing a tissue-level signal that pain scores cannot deliver.

### 11.3. Wearable Inertial Sensors

Wearable inertial measurement units (IMUs) and smartphone-based motion-capture systems allow continuous, ecologically valid measurement of shoulder kinematics during everyday tasks. Compared with clinic-based goniometry, IMU data capture compensatory scapulothoracic motion, multi-planar coupling, and movement velocity—dimensions that single-plane ROM measurement misses [17]. Validation studies report good concurrent validity and inter- and intra-rater reliability of IMU-based shoulder ROM against goniometry (intraclass correlation coefficients broadly in the 0.6–0.97 range across movements), and a systematic review and meta-analysis found that digital ROM devices compare closely with universal goniometry (pooled mean bias near zero) [37,38]. In AC specifically, integrated wearable-plus-virtual-reality systems have been used to deliver and quantify goal-directed shoulder rehabilitation, with data-mined motor ingredients correlating with clinical assessment [54], and machine-learning analyses of IMU signals have demonstrated automated identification of frozen-shoulder kinematics from daily life tasks [55]. As with SWE, sensor model, sampling frequency, and validation reference vary across studies and should be specified in primary reports. Combined with elastography and high-resolution ultrasound, these tools shift outcome assessment from periodic clinic snapshots toward continuous, biologically grounded measurement.

## 12. Limitations of Current Evidence and Research Priorities

Several gaps remain, and they bound the strength of the recommendations above. Head-to-head comparative trials among HA, prolotherapy, and PRP are scarce, and existing comparisons are frequently single-center, of modest sample size, open-label, heterogeneous in protocol, and limited in follow-up; none of the pivotal cited trials reported an a priori sample-size calculation except for the HA-versus-rehabilitation trial [34], and effect estimates from small single-center studies are likely to shrink upon multicenter replication. The optimal combination of intra-articular and perineural strategies—specifically whether routine combined intra-articular regenerative injection plus SSN hydrodissection improves outcomes over intra-articular injection alone—remains undefined. Several recommendations rest partly on evidence extrapolated from adjacent pathologies (dextrose from rotator cuff tendinopathy; perineural hydrodissection mechanisms from entrapment neuropathies; chondrotoxicity of steroid from knee osteoarthritis), and although we have flagged these extrapolations at the point of recommendation, they remain a source of uncertainty. Standardization of PRP preparation (platelet concentration, leukocyte content, activation) and of dextrose protocols (concentration, volume, frequency) is overdue [44]. Long-term outcomes (≥12 months), particularly in diabetic and refractory subgroups, are insufficiently reported, and the integration of objective monitoring tools (SWE, wearables) into trial endpoints is in its infancy. Future research should prioritize multicenter randomized trials with stratification by disease phase and diabetic status, harmonized and fully reported injection protocols, blinded outcome assessment, and biologically anchored objective outcomes. Trials directly comparing the proactive non-steroidal-first paradigm against the corticosteroid-first paradigm endorsed by the major reviews [8,9,10] are particularly needed.

A specific limitation deserves explicit acknowledgment. The phase-specific algorithm proposed in Section 9 and Figure 3 is a clinical framework, not a formally evidence-graded guideline. While each intervention node is supported by published randomized or meta-analytic data—or, where it is not, is flagged as extrapolated or case-level—the integration of these interventions into a unified step-up sequence, and the chosen reassessment timepoints (4–6 and 12–16 weeks), reflect the authors’ single-center clinical practice and have not been validated prospectively as a single protocol. A pragmatic implementation trial comparing this proactive non-steroidal-first paradigm against the prevailing corticosteroid-first standard would be a logical next step.

## 13. Conclusions

Adhesive capsulitis need not be regarded as a condition that patients must simply endure. The evidence summarized here supports, in appropriately selected patients, consideration of a more active stance than the one currently endorsed by the major reviews [8,9,10]. In the randomized comparisons available to date, ultrasound-guided regenerative injectates—HA, hypertonic dextrose prolotherapy, and PRP—appear non-inferior to corticosteroids for medium-term pain, disability, and ROM, and they avoid the diabetic and tissue-toxicity costs that limit repeated steroid use; they may also help prevent the slow build-up of organized capsular adhesions. Perineural hydrodissection of the suprascapular—and, with weaker evidence, the axillary and subscapular—nerves adds a distinct neuromodulation pathway that can broaden what patients tolerate in early rehabilitation. Used within a phase-specific framework—pain-controlling injection in the freezing phase, structured graded rehabilitation through the frozen-to-thawing transition—these strategies may compress recovery toward the three-to-six-month range, a hypothesis that remains to be confirmed prospectively. The qualifier is essential: injection is the catalyst, and rehabilitation performs most of the functional work. Wearable inertial sensors and shear-wave elastography are beginning to make that rehabilitation more individualized by supplying objective data that were not previously available, although their validation in AC is still early. The outstanding tasks—standardizing protocols, conducting head-to-head and multicenter trials, and expanding training in ultrasound-guided technique—are substantial. Until they are completed, the available evidence supports a step-up, ultrasound-guided, non-steroidal-first approach combined with structured rehabilitation as a clinically defensible option for adhesive capsulitis, offered with appropriate acknowledgement of its current evidentiary limits.

## 14. A Practical Outlook

Three directions seem most worth pursuing. The first is better matching of patients to treatment. Not every frozen shoulder is the same, yet current practice still tends to treat them as if they were. Capsular stiffness on shear-wave elastography, glycemic status, prior injection history, and the dominant pain pattern (capsular versus neuropathic) are all variables that could inform whether a given patient receives HA, PRP, perineural hydrodissection, or some combination, but none is yet routinely used to stratify treatment. Closing this gap requires not new technology but the discipline to apply what is already available.

The second is rebalancing the partnership with the patient. As wearable sensors enter daily rehabilitation, patients are increasingly able to see their own recovery in close to real time—when abduction improves, when scapular compensation lessens, or when a home-exercise day has been missed. That visibility can change the clinical conversation, making recovery something the patient participates in rather than something measured only at the clinic. For a condition whose chief threat is loss of autonomy in daily life, this shift is clinically meaningful.

The third is honest comparative evidence. Most current data come from small trials of single agents against placebos or corticosteroids. What patients and clinicians need are head-to-head trials between regenerative agents, between intra-articular and perineural strategies, and between proactive non-steroidal protocols and the corticosteroid-first paradigm still endorsed by the major reviews [8,9,10]. Generating this evidence is slow and resource-intensive, but it is the only way to move the field beyond preference-based prescribing. None of these directions requires waiting for a technological breakthrough; each requires applying existing tools—ultrasound guidance, regenerative injectates, perineural hydrodissection, structured rehabilitation, and increasingly objective monitoring—with greater consistency, greater transparency about what remains unknown, and closer attention to the function each patient is trying to recover.

## Data Availability

No new data were created or analyzed in this study. Data sharing is not applicable to this article.
